# Plasma and urinary sTREM-1 as biomarkers of sepsis-associated acute kidney injury in critically Ill children

**DOI:** 10.1007/s00431-026-07202-z

**Published:** 2026-06-30

**Authors:** Sondos M. Magdy, Tarek AAbd El-Gawaad, Fatma A. Hussein, Mohamed S. El-Farsy

**Affiliations:** 1 Pediatric Intensive Care Unit, Children’s Hospital, Ain Shams University, Cairo, Egypt; 2 Pediatric Nephrology Department, Children’s Hospital, Ain Shams University, Cairo, Egypt

**Keywords:** Sepsis-associated acute kidney injury, STREM-1, Pediatric sepsis, Biomarkers, PSOFA

## Abstract

Early identification of sepsis-associated acute kidney injury (SA-AKI) is important to enable timely supportive management and reduce further renal injury. However, conventional diagnostic criteria for acute kidney injury (AKI), based on serum creatinine and urine output, may delay early detection. Soluble triggering receptor expressed on myeloid cells-1 (sTREM-1) has emerged as a potential biomarker in septic conditions. To evaluate the diagnostic value of plasma and urinary soluble triggering receptor expressed on myeloid cells-1 (sTREM-1) in children with sepsis-associated acute kidney injury. This prospective observational cohort study included 50 children with sepsis admitted to the Pediatric Intensive Care Unit, Children’s Hospital, Ain Shams University, Cairo, Egypt, between October 2023 and March 2024. Plasma and urinary sTREM-1 levels were measured at the time of sepsis diagnosis according to pediatric Sequential Organ Failure Assessment (pSOFA) criteria. Patients were prospectively followed for the development of SA-AKI, and in those who developed AKI according to pediatric Risk, Injury, Failure, Loss, End-stage kidney disease (pRIFLE) criteria, sTREM-1 levels were reassessed at the time of AKI diagnosis. The median age of the study population was 8.5 months (range 3–24), with a slight female predominance (54%). Plasma sTREM-1 levels were significantly higher in children who developed SA-AKI than in septic children without AKI [222.7 (165.2–325.9) vs 130.3 (85.1–206.2) pg/mL, *p* = 0.006]. Receiver operating characteristic (ROC) analysis showed an area under the curve (AUC) of 0.729, with a cutoff value of 159.6 pg/mL, yielding 80% sensitivity and 60% specificity. Urinary sTREM-1 levels were also significantly higher in children who developed SA-AKI [116.3 (53.9–197.3) vs 68.5 (46.7–112.2) pg/mL, *p* = 0.045]. ROC analysis showed an AUC of 0.666, with a cutoff value of 62.2 pg/mL, yielding 72% sensitivity and 48% specificity. *Conclusion*: Both plasma and urinary sTREM-1 levels were significantly elevated in children who developed sepsis-associated acute kidney injury. Measurement at sepsis diagnosis may aid early risk stratification, while repeat assessment at AKI diagnosis may provide additional prognostic information.

**Table:** 

**What is Known:** • *Early recognition of sepsis-associated acute kidney injury in children is challenging, and conventional diagnostic markers may be delayed. sTREM-1 is an inflammatory biomarker with potential value in sepsis-related organ injury.*	
**What this study adds:** *• Baseline plasma and urinary sTREM-1 levels were higher in septic children who later developed sepsis-associated acute kidney injury, and both rose further after AKI occurrence. Urinary sTREM-1 also showed strong prognostic value for mortality in children with sepsis-associated acute kidney injury.*

**What is Known:**

• *Early recognition of sepsis-associated acute kidney injury in children is challenging, and conventional diagnostic markers may be delayed. sTREM-1 is an inflammatory biomarker with potential value in sepsis-related organ injury.*

**What this study adds:**

*• Baseline plasma and urinary sTREM-1 levels were higher in septic children who later developed sepsis-associated acute kidney injury, and both rose further after AKI occurrence. Urinary sTREM-1 also showed strong prognostic value for mortality in children with sepsis-associated acute kidney injury.*

## Introduction

Sepsis is a life-threatening condition caused by a dysregulated host response to infection and remains a major cause of morbidity and mortality in critically ill children. Among the organs affected during sepsis, the kidney is particularly vulnerable, and acute kidney injury (AKI) is one of the most frequent and serious complications. Sepsis-associated acute kidney injury (SA-AKI) contributes substantially to prolonged pediatric intensive care unit (PICU) stay, need for organ support, and increased mortality. Pediatric studies have demonstrated that AKI occurs frequently in critically ill children and is associated with significantly worse outcomes, particularly in severe stages [1, 2].

The pathophysiology of SA-AKI is complex and multifactorial. In contrast to the traditional concept that AKI primarily results from renal hypoperfusion, current evidence suggests that inflammatory injury, endothelial dysfunction, microcirculatory disturbances, tubular epithelial cell injury, and immune dysregulation all contribute to the development of renal injury during sepsis. Activation of pathogen-associated and damage-associated molecular patterns triggers innate immune responses and cytokine release, leading to alterations in renal microcirculation and tubular dysfunction that may precede measurable changes in conventional renal function markers [3–6].

Despite the widespread use of Kidney Disease Improving Global Outcomes (KDIGO) criteria, the diagnosis of AKI still relies mainly on serum creatinine levels and urine output. Both markers have important limitations in the early phase of kidney injury. Serum creatinine is a delayed indicator and may not rise until substantial renal damage has already occurred, while urine output may be affected by hemodynamic status, fluid administration, and diuretic therapy. Consequently, there is increasing interest in identifying novel biomarkers that can allow earlier and more accurate detection of kidney injury in septic patients [7, 8].

Triggering receptor expressed on myeloid cells-1 (TREM-1) is an inflammatory receptor expressed on neutrophils, monocytes, macrophages, and other innate immune cells. It amplifies inflammatory responses initiated through toll-like receptor and nod-like receptor signaling pathways. The soluble form of this receptor, soluble TREM-1 (sTREM-1), can be detected in body fluids and has been investigated as a biomarker in sepsis and other infectious diseases. Because SA-AKI is strongly linked to inflammatory activation, sTREM-1 may represent a promising biomarker for the early detection of kidney injury in septic patients [9–12].

Therefore, this study was designed to evaluate the diagnostic performance of plasma and urinary sTREM-1 for identifying sepsis-associated acute kidney injury in children admitted to the pediatric intensive care unit.

## Methods

### Study design and population

This prospective observational cohort study was conducted in the Pediatric Intensive Care Unit (PICU) of the Children’s Hospital, Ain Shams University, Cairo, Egypt, between October 2023 and March 2024. Fifty children aged 1 month to 15 years who fulfilled criteria for sepsis or septic shock were enrolled at admission.

Sepsis was defined as suspected or confirmed infection associated with organ dysfunction and a pediatric Sequential Organ Failure Assessment (pSOFA) score greater than 2 [1]. Septic shock was defined as the requirement for vasopressor support to maintain mean arterial pressure above age-adjusted thresholds with serum lactate levels exceeding 2 mmol/L despite adequate fluid resuscitation.

At the time of diagnosis of sepsis or septic shock, according to pSOFA criteria, baseline plasma and urinary soluble triggering receptor expressed on myeloid cells-1 (sTREM-1) levels were measured in all patients. Patients were then prospectively followed for the development of sepsis-associated acute kidney injury (SA-AKI) during their PICU stay.

Acute kidney injury was defined according to the pediatric Risk, Injury, Failure, Loss, End-stage kidney disease (pRIFLE) criteria [13], based on changes in estimated glomerular filtration rate (eGFR), serum creatinine level, or urine output. Estimated glomerular filtration rate was calculated using the bedside Schwartz Eq [14].

We used pRIFLE because it was the prespecified classification system in the original study protocol and allowed incorporation of estimated glomerular filtration rate using the bedside Schwartz equation. However, we recognize that KDIGO is currently the more widely adopted definition for pediatric AKI and may improve comparability across contemporary studies.

According to subsequent AKI occurrence, patients were classified into those who developed SA-AKI (n = 25) and those who did not (n = 25). In patients who developed SA-AKI, plasma and urinary sTREM-1 levels were reassessed at the time of AKI diagnosis to evaluate dynamic biomarker changes.

Patients were excluded if they had pre-existing chronic kidney disease, chronic renal failure, renal transplantation, or underlying renal disorders such as nephrolithiasis, nephrotic syndrome, or tubulopathies. Patients with chronic inflammatory diseases, including systemic lupus erythematosus, inflammatory bowel disease, or rheumatoid arthritis, were also excluded.

### Clinical assessment

All patients underwent comprehensive clinical evaluation on admission. Demographic data, including age, sex, and consanguinity, were recorded. The underlying diagnosis and severity of illness were assessed using the pediatric Sequential Organ Failure Assessment (pSOFA) score.

Vital signs, including respiratory rate, heart rate, blood pressure, and body temperature, were documented. Anthropometric measurements, including weight and height, were obtained. Medication history, including the use of vasoactive drugs, was also recorded.

### Laboratory investigations

Laboratory investigations included complete blood count, C-reactive protein, venous blood gas analysis, and serum lactate. Renal function was assessed using serum urea and creatinine levels. Serum electrolytes, including sodium, potassium, calcium, and phosphorus, were measured. Additional investigations included alkaline phosphatase, routine urine analysis, and blood culture with sensitivity testing.

Blood and urine samples were collected for measurement of soluble triggering receptor expressed on myeloid cells-1 (sTREM-1). Levels of sTREM-1 were determined using a commercially available enzyme-linked immunosorbent assay kit (Human sTREM-1 ELISA kit, Bioassay Technology Laboratory, Shanghai, China; Cat. No. E0310Hu) according to the manufacturer’s instructions.

### Sample size calculation

Sample size was calculated using PASS software (version 11). Based on previously published data, [15] a minimum sample size of 45 patients was required to achieve 99% power at a 5% significance level for detecting the diagnostic performance of sTREM-1 in sepsis-associated acute kidney injury. To account for potential dropout, the sample size was increased to 50 patients.

### Ethical considerations

The study protocol was approved by the Research Ethics Committee of the Faculty of Medicine, Ain Shams University (FMASU MS 605/2023; FWA 000017585). Written informed consent was obtained from the legal guardians of all participants prior to enrollment, in accordance with the Declaration of Helsinki.

### Statistical analysis

Statistical analysis was performed using IBM SPSS Statistics for Windows, version 22.0 (IBM Corp., Armonk, NY, USA). Quantitative variables were expressed as mean ± standard deviation for normally distributed data and median with interquartile range for non-normally distributed data. Qualitative variables were presented as numbers and percentages.

Comparisons between groups were performed using the independent Student’s t-test or Mann–Whitney U test, as appropriate. Categorical variables were compared using the Chi-square test or Monte Carlo correction.

Receiver operating characteristic (ROC) curve analysis was used to evaluate the diagnostic performance of sTREM-1, and the area under the curve (AUC) was calculated. Sensitivity and specificity were determined at the optimal cutoff value. Correlations between plasma and urinary sTREM-1 and selected demographic, clinical, and laboratory variables were assessed using Spearman’s rank correlation coefficient. Among children who developed SA-AKI, comparisons of plasma and urinary sTREM-1 levels across pRIFLE severity categories were performed using the Kruskal–Wallis test. A *p*-value < 0.05 was considered statistically significant.

## Results

A total of 50 children with sepsis were enrolled and underwent baseline plasma and urinary sTREM-1 measurement at the time of sepsis diagnosis. During follow-up, 25 patients (50%) developed sepsis-associated acute kidney injury (SA-AKI), whereas 25 did not.

Baseline demographic and anthropometric characteristics are summarized in Table 1. Children who subsequently developed SA-AKI were significantly younger than those who did not develop AKI (median age 0.42 vs 1.0 years, *p* = 0.048). They also had lower median height (62 vs 75 cm, *p* = 0.03) and lower median weight (6.4 vs 9.5 kg, *p* = 0.04). However, no significant differences were observed between groups regarding sex distribution, height percentile, weight percentile, or baseline pSOFA score.

**Table 1:** Baseline demographic and anthropometric characteristics of enrolled septic children stratified by subsequent AKI development

Variable		Developed SA-AKI (*n* = 25)	Did not develop AKI (*n* = 25)	*p*-value
Sex, n (%)*	Male	10 (40)	13 (52)	0.73
	Female	15 (60)	12 (48)	
Age, years, median (IQR)^#^		0.42 (0.23–1.25)	1.00 (0.67–3.75)	0.048
Height, cm, median (IQR)^#^		62 (55–77.5)	75 (64.5–100)	0.03
Height percentile, median (IQR)^#^		19 (2.35–64.4)	36.5 (12.65–82.6)	0.09
Weight, kg, median (IQR)^#^		6.4 (5–9)	9.5 (7–14.75)	0.04
Weight percentile, median (IQR)^#^		9.6 (1.7–33.85)	16.4 (2.9–46.75)	0.27
pSOFA score, median (IQR)^#^		8 (6–11.5)	7 (5–14)	0.85

*AKI* Acute kidney injury, *IQR* Interquartile range, *pSOFA* Pediatric sequential organ failure assessment, # Mann Whitney test, *Chi square test, *p*-value < 0.05: Significant

Baseline laboratory and biomarker findings are shown in Table 2. No significant differences were observed between groups regarding baseline CRP, lactate, serum creatinine, or serum urea. In contrast, serum uric acid was significantly higher in children who subsequently developed SA-AKI. Baseline plasma and urinary sTREM-1 levels were also significantly higher in patients who later developed SA-AKI. Median plasma sTREM-1 was 222.7 pg/mL (IQR 165.2–325.9) in children who developed AKI compared with 130.3 pg/mL (IQR 85.05–206.2) in those who did not (*p* = 0.006), while median urinary sTREM-1 was 116.3 pg/mL (IQR 53.93–197.3) versus 68.5 pg/mL (IQR 46.66–112.15), respectively (*p* = 0.045).

**Table 2:** Baseline laboratory and biomarker findings according to subsequent development of sepsis-associated acute kidney injury

Variable	Developed SA-AKI (*n* = 25)	Did not develop AKI (*n* = 25)	*p*-value
CRP, mg/L, median (IQR)^#^	53 (26.65–98.35)	60 (23.65–191.35)	0.29
Lactate, mmol/L, median (IQR)^#^	1.82 (1.45–2.1)	1.9 (1.2–2.3)	0.83
Serum creatinine, mg/dL, median (IQR)^#^	0.4 (0.2–0.57)	0.4 (0.3–0.66)	0.37
Serum urea, mg/dL, median (IQR)^#^	24 (22–28.5)	25 (22–29)	0.58
Serum uric acid, mg/dL, mean ± SD^+^	**3.39 ± 1.19**	**2.44 ± 0.67**	**0.001**
Plasma sTREM-1, pg/mL, median (IQR)^#^	**222.7 (165.2–325.9)**	**130.3 (85.05–206.2)**	**0.006**
Urine sTREM-1, pg/mL, median (IQR)^#^	**116.3 (53.93–197.3)**	**68.5 (46.66–112.15)**	**0.045**

*AKI* Acute kidney injury, *CRP* C reactive protein, *sTREM* Soluble triggering receptor expressed on myeloid cells, *UA* Uric acid, *SD* Standard deviation, + Independent student t test, # Mann Whitney test, *p*-value < 0.05: Significant

**Fig. 1:**
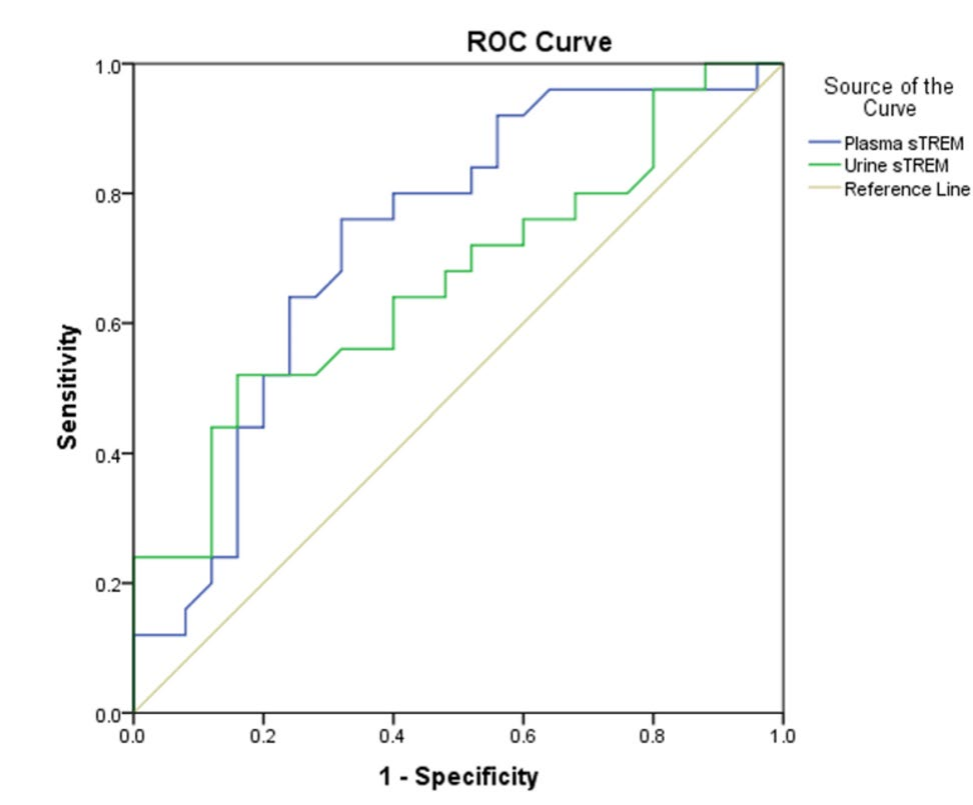
ROC curves of plasma and urinary sTREM-1 for sepsis-associated AKI

Receiver operating characteristic analysis showed that plasma sTREM-1 had moderate discriminatory ability for identifying SA-AKI, with an area under the curve (AUC) of 0.729 (95% CI 0.59–0.87; *p* = 0.006). At a cutoff value of 159.6 pg/mL, plasma sTREM-1 yielded 80% sensitivity and 60% specificity. Urinary sTREM-1 showed modest discriminatory performance, with an AUC of 0.666 (*p* = 0.045). At a cutoff value of 62.2 pg/mL, urinary sTREM-1 yielded 72% sensitivity and 48% specificity (Fig. 1).

Among patients who developed SA-AKI, both plasma and urinary sTREM-1 levels increased significantly after AKI occurrence (Table 3). Median plasma sTREM-1 increased from 222.7 pg/mL (IQR 165.2–325.9) before AKI to 386.4 pg/mL (IQR 294.55–456.15) after AKI development (*p* = 0.001). Median urinary sTREM-1 also increased from 116.3 pg/mL (IQR 53.93–197.3) to 214 pg/mL (IQR 150.5–314.5) after AKI development (*p* = 0.001).

**Table 3:** Changes in plasma and urinary sTREM-1 levels before and after development of sepsis-associated acute kidney injury

Variable	Pre-AKI	Post-AKI	*p*-value
Plasma sTREM-1, pg/mL, median (IQR)	222.7 (165.2–325.9)	386.4 (294.55–456.15)	0.001
Urine sTREM-1, pg/mL, median (IQR)	116.3 (53.93–197.3)	214 (150.5–314.5)	0.001

*AKI* Acute kidney injury, *sTREM* Soluble triggering receptor expressed on myeloid cells, $: Wilcoxon signed-rank test, *p*-value < 0.05: Significant

Overall mortality was 24%. Plasma and urinary sTREM-1 levels were significantly higher in non-survivors than in survivors (Table 4). Median plasma sTREM-1 was 234.2 pg/mL (IQR 108.82–400.65) in non-survivors compared with 170.4 pg/mL (IQR 88.9–243.9) in survivors (*p* = 0.001). Median urinary sTREM-1 was 183 pg/mL (IQR 115.4–253.1) in non-survivors compared with 61.8 pg/mL (IQR 45.46–111.2) in survivors (*p* = 0.001).

**Table 4:** Plasma and urinary sTREM-1 levels according to survival outcome

Variable	Survivors (*n* = 38)	Non-survivors (*n* = 12)	*p*-value
Plasma sTREM-1, pg/mL, median (IQR)^#^	170.4 (88.9–243.9)	234.2 (108.82–400.65)	0.001
Urine sTREM-1, pg/mL, median (IQR)^#^	61.8 (45.46–111.2)	183 (115.4–253.1)	0.001

*IQR* Interquartile range, *sTREM* Soluble triggering receptor expressed on myeloid cells, #: Mann Whitney test, *p*-value < 0.05: Significant

Within the SA-AKI subgroup, sTREM-1 levels were consistently higher in non-survivors than in survivors both before and after AKI occurrence. Before AKI occurrence, median plasma sTREM-1 was 311.7 pg/mL in non-survivors versus 213.5 pg/mL in survivors (*p* = 0.05), while median urinary sTREM-1 was 220.85 pg/mL versus 68.62 pg/mL, respectively (*p* = 0.001). After AKI development, both plasma and urinary sTREM-1 remained significantly higher in non-survivors, with median plasma levels of 455.6 versus 338.85 pg/mL (*p* = 0.027) and median urinary levels of 223 versus 158 pg/mL (*p* = 0.013).

**Fig. 2:**
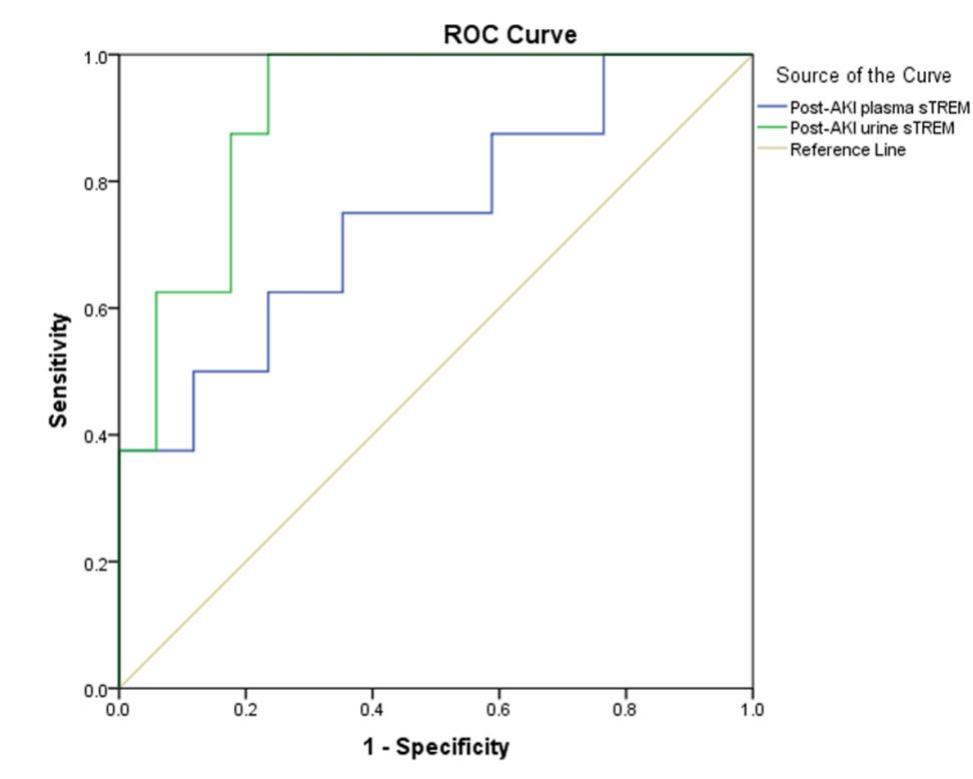
ROC curves of plasma and urinary sTREM-1 for mortality

Receiver operating characteristic analysis was also performed to assess the ability of plasma and urinary sTREM-1 to predict mortality among children with SA-AKI. Plasma sTREM-1 showed moderate predictive performance, with an AUC of 0.74 (95% CI 0.53–0.96; *p* = 0.05) at a cutoff value of 228.45 pg/mL, yielding 75% sensitivity and 64.6% specificity. Urinary sTREM-1 showed excellent predictive performance, with an AUC of 0.91 (95% CI 0.80–1.00; *p* = 0.001) at a cutoff value of 117.3 pg/mL, yielding 100% sensitivity and 76.5% specificity (Fig. 2).

Among children who developed SA-AKI, no significant monotonic correlation was observed between pRIFLE severity stage and baseline or post-AKI plasma or urinary sTREM-1 levels. However, baseline urinary sTREM-1 differed significantly across pRIFLE categories on Kruskal–Wallis testing (*p* = 0.039).

Correlation analysis showed no significant association between plasma or urinary sTREM-1 and CRP, lactate, or pSOFA score. Plasma and urinary sTREM-1 were significantly positively correlated (*r *= 0.35, *p* = 0.013). Plasma sTREM-1 also showed significant correlations with serum potassium (*r* = 0.35, *p* = 0.01) and several anthropometric variables.

## Discussion

In this prospective cohort of critically ill children with sepsis, both plasma and urinary sTREM-1 levels were higher in patients who subsequently developed sepsis-associated acute kidney injury (SA-AKI). Among children who developed SA-AKI, both biomarkers increased further after AKI occurrence, and higher levels were also associated with mortality, particularly for urinary sTREM-1. These findings support a potential role for sTREM-1 as an adjunctive biomarker for the identification of SA-AKI and for risk stratification in affected patients.

The observed age difference between groups should be interpreted cautiously. Children who developed SA-AKI were significantly younger and had lower absolute height and weight values; however, height and weight percentiles did not differ significantly, suggesting that these anthropometric differences were primarily age-related rather than reflective of a distinct nutritional pattern. Younger age may nevertheless identify a more vulnerable subgroup with lower physiologic reserve and greater susceptibility to sepsis-related renal injury. This is consistent with pediatric AKI literature showing a substantial burden of AKI in younger children, although age-related patterns vary across settings and underlying diagnoses [16, 17].

In the present study, there were no significant baseline differences between groups regarding CRP, lactate, serum creatinine, or serum urea, whereas serum uric acid was significantly higher in children who subsequently developed SA-AKI. The lack of significant differences in conventional renal indices at baseline is not unexpected, because patients were grouped according to later AKI development and one of the main objectives was to determine whether sTREM-1 could identify renal injury earlier than routine parameters. The higher uric acid level may reflect increased metabolic stress, altered renal handling, or greater renal vulnerability in the SA-AKI group. A similar association between elevated uric acid and AKI has been reported in pediatric patients [18].

A major finding of this study was the significant elevation of baseline plasma sTREM-1 in children who later developed SA-AKI, with a further significant increase after AKI occurrence. Plasma sTREM-1 also showed moderate discriminatory performance for identifying SA-AKI. These findings are broadly consistent with Elbaz et al., who reported significantly higher plasma sTREM-1 levels in Egyptian children with sepsis-associated AKI compared with septic children without AKI, although the discriminatory performance in their cohort was higher than in ours. [19] This difference may reflect variation in patient characteristics, severity of illness, sample size, or timing of biomarker measurement. Accordingly, our findings support the use of plasma sTREM-1 as a potentially useful adjunctive biomarker, while also suggesting that it should not yet be considered a standalone diagnostic test.

Urinary sTREM-1 was also significantly higher at baseline in patients who subsequently developed SA-AKI and increased further after AKI occurrence. Although its discriminatory ability for identifying SA-AKI was lower than that of plasma sTREM-1 in our study, the dynamic rise after AKI development suggests that urinary sTREM-1 may reflect ongoing renal inflammatory injury. Taken together, the plasma and urinary measurements indicate that sTREM-1 may capture complementary aspects of systemic and renal inflammatory activation.

Both plasma and urinary sTREM-1 levels were significantly higher in non-survivors than in survivors. Within the SA-AKI subgroup, elevated pre-AKI and post-AKI biomarker levels were consistently associated with mortality, particularly for urinary sTREM-1. Plasma sTREM-1 showed moderate prognostic performance, whereas urinary sTREM-1 demonstrated excellent predictive performance for mortality. These findings suggest that urinary sTREM-1 may better reflect the severity of renal inflammatory injury and may therefore have greater prognostic utility than plasma levels in children with SA-AKI. This is consistent with adult studies showing higher circulating sTREM-1 levels in septic non-survivors and good prognostic performance of urinary sTREM-1 for adverse outcomes [20, 21].

Several mechanisms may explain the observed association between elevated sTREM-1 and SA-AKI. sTREM-1 is released from membrane-bound TREM-1 following inflammatory cell activation and proteolytic cleavage, and higher levels may therefore indicate more intense inflammatory signaling and tissue injury [15]. Persistent exposure to microbial products and inflammatory mediators during sepsis may amplify TREM-1 signaling, enhance endothelial dysfunction, and promote leukocyte migration, thereby sustaining both systemic inflammation and renal injury [20, 22]. In addition, recent work suggests that TREM-1 may contribute more directly to kidney damage through cytokine amplification, oxidative stress, recruitment of inflammatory cells, and effects on apoptosis and autophagy in renal tissue [23]. These mechanisms support the biologic plausibility of sTREM-1 as both a biomarker and a possible mediator of SA-AKI severity.

We did not observe a significant monotonic correlation between sTREM-1 levels and AKI severity stage, although baseline urinary sTREM-1 differed across pRIFLE categories. This finding should be interpreted cautiously because the subgroup sample size was small and the stage distribution was uneven.

An additional finding was that neither plasma nor urinary sTREM-1 showed significant correlation with CRP, lactate, or pSOFA score. This suggests that sTREM-1 may reflect a biological process not fully captured by conventional inflammatory markers or global severity scoring. In contrast, the significant positive correlation between plasma and urinary sTREM-1 supports internal consistency of the biomarker profile.

From a clinical perspective, our findings suggest that plasma and urinary sTREM-1 are most informative when measured at the time of diagnosis of sepsis or septic shock to identify children at increased risk of subsequent SA-AKI. In children who develop SA-AKI, repeat measurement at the time of AKI diagnosis may provide additional prognostic information, particularly for urinary sTREM-1. However, given the modest diagnostic performance for baseline SA-AKI identification, sTREM-1 should currently be regarded as an adjunctive biomarker to clinical assessment and conventional renal monitoring rather than a replacement for established diagnostic criteria.

This study has some limitations. It was conducted at a single center and included a relatively small sample size, which may limit generalizability and reduce precision of effect estimates. In addition, there was no healthy control group, and sTREM-1 was not compared with other established AKI biomarkers such as NGAL or KIM-1. However, the prospective design, baseline measurement of both plasma and urinary sTREM-1 in all septic children, and repeated assessment after AKI development strengthen the interpretation of the findings. Another limitation is that AKI was classified using pRIFLE rather than KDIGO criteria. Although pRIFLE remains clinically informative in pediatric practice, KDIGO is currently more widely used and may provide better comparability with recent pediatric AKI studies. Because this was an observational cohort study, causal relationships cannot be inferred.

In conclusion, both plasma and urinary sTREM-1 were associated with subsequent development of sepsis-associated acute kidney injury in critically ill children. Measurement at the time of diagnosis of sepsis or septic shock may help identify children at higher risk of SA-AKI, while repeat assessment at the time of AKI diagnosis, particularly of urinary sTREM-1, may aid prognostic stratification. Larger multicenter studies are needed before routine clinical implementation can be recommended.

## Data Availability

The datasets used and/or analysed during the current study are available from the corresponding author on reasonable request.

## Abbreviations

AKI: Acute kidney injury
AUC: Area under the curve
CI: Confidence interval
CRP: C-reactive protein
eGFR: Estimated glomerular filtration rate
FMASU: Faculty of Medicine, Ain Shams University
FWA: Federal Wide Assurance
IQR: Interquartile range
KDIGO: Kidney Disease: Improving Global Outcomes
PICU: Pediatric intensive care unit
pRIFLE: Pediatric Risk, Injury, Failure, Loss, End-stage kidney disease
pSOFA: Pediatric Sequential Organ Failure Assessment
ROC: Receiver operating characteristic
SA-AKI: Sepsis-associated acute kidney injury
SD: Standard deviation
sTREM-1: Soluble triggering receptor expressed on myeloid cells-1
TREM-1: Triggering receptor expressed on myeloid cells-1
